# Total Analysis of the Major Secoiridoids in Extra Virgin Olive Oil: Validation of an UHPLC-ESI-MS/MS Method

**DOI:** 10.3390/antiox10040540

**Published:** 2021-03-30

**Authors:** Julián Lozano-Castellón, Anallely López-Yerena, Alexandra Olmo-Cunillera, Olga Jáuregui, Maria Pérez, Rosa Mª Lamuela-Raventós, Anna Vallverdú-Queralt

**Affiliations:** 1 Department of Nutrition, Food Science and Gastronomy, XaRTA, Institute of Nutrition and Food Safety (INSA-UB), School of Pharmacy and Food Sciences, University of Barcelona, 08028 Barcelona, Spain; julian.lozano@ub.edu (J.L.-C.); naye.yerena@gmail.com (A.L.-Y.); alexandra.olmo@ub.edu (A.O.-C.); mariaperez@ub.edu (M.P.); lamuela@ub.edu (R.M.L.-R.); 2 CIBER Physiopathology of Obesity and Nutrition (CIBEROBN), Institute of Health Carlos III, 28029 Madrid, Spain; 3 Center of Scientific and Technological, University of Barcelona (CCiTUB), 08028 Barcelona, Spain; ojauregui@ccit.ub.edu; 4 CIBER Fragilidad y Envejecimiento Saludable (CIBERfes), Instituto de Salud Carlos III, 18100 Barcelona, Spain; 5 Laboratory of Organic Chemistry, Faculty of Pharmacy and Food Sciences, University of Barcelona, 08028 Barcelona, Spain

**Keywords:** EVOO analysis, polyphenols, Mediterranean diet, oleocanthal, oleacein, HPLC, mass spectrometry

## Abstract

Extra virgin olive oil (EVOO), one of the key foods of the Mediterranean diet, is distinguished by its high content of nutritional and antioxidant compounds compared to other vegetable oils. During EVOO production, the major secoiridoids of EVOO, oleacein, oleocanthal, ligstroside, and oleuropein aglycones, undergo a series of transformations to open- and closed-structure forms. The resulting mixture of compounds can become more complex during the analytical procedure, due to the keto-enol tautomerism of the open forms and their interaction with polar solvents, and therefore more challenging to analyze. Employing the same extraction method used to analyze the other EVOO phenolic compounds, we report here a simple UHPLC-ESI-MS/MS procedure for the quantification of those secoiridoids that is able to co-elute the different isomers of each compound. The method was validated following AOAC guidelines, and the matrix effect and recoveries were within satisfactory limits.

## 1. Introduction

Extra virgin olive oil (EVOO) is the main fat of the Mediterranean Diet and its consumption is related to some health benefits as protection against cancer [1], type II diabetes [2], neurodegeneration [3], cardiovascular diseases, and reduces total mortality [4]. Those are attributed to its fatty acid profile, rich in mono-unsaturated fatty acids, and to its non-saponifiable fraction, rich in antioxidants [5]. The non-saponifiable fraction is composed by lipophilic compounds as vitamins, mainly α-tocopherol; and carotenoids, mostly lutein and β-carotene; and a more hydrophilic molecules as phenolic compounds, above all, secoiridoids (SEC). This fraction, in addition to its health benefits, is the responsible of EVOO high appreciated taste. The volatiles compounds are responsible of its aroma, giving to it from notes of green fruit to notes of banana. Moreover, the phenolic compounds are well correlated with bitter, fruity, green, and pungent EVOO taste descriptors [6].

**Figure 1:**
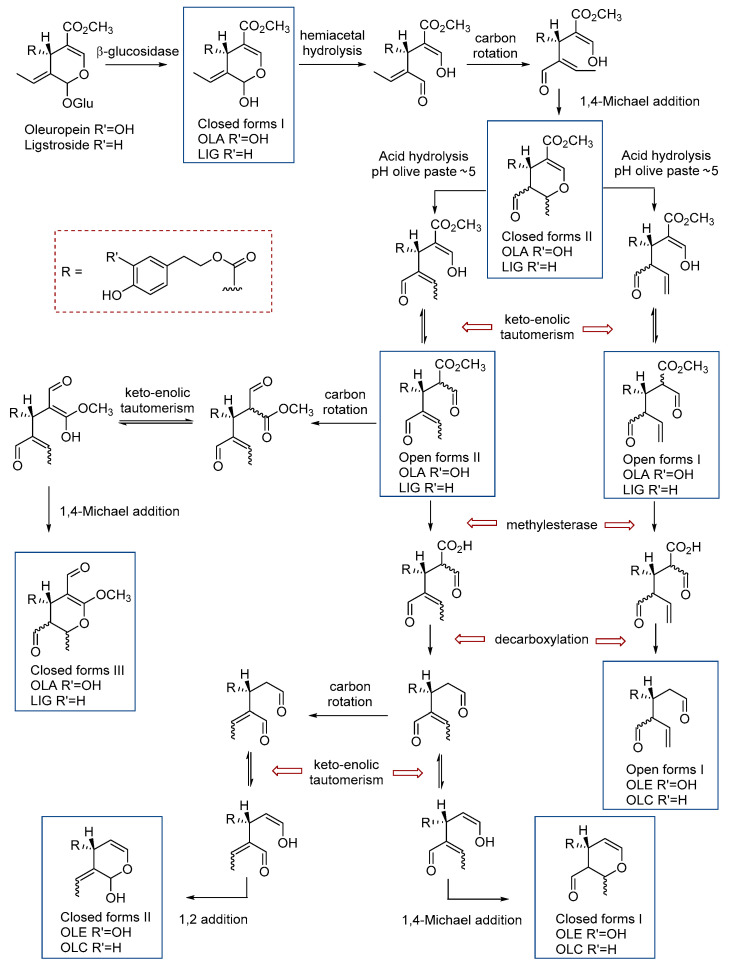
Conversion of Oleuropein and Ligstroside to oleuropein aglycone (OLA), oleacein (OLE), ligstroside aglycone (LIG), and oleocanthal (OLC) during oil extraction. Adapted from Abbattista et al., 2019 [10] and adding the closed forms I and II for OLC and OLE, which are possible artifacts.

SEC represent 70–90% of the total phenolic compounds in EVOO [7]. Among the major SEC in olive drupes are oleuropein and ligstroside [8,9], which evolve to their constituent aglycones of oleuropein (OLA) and ligstroside (LIG) during EVOO production. The hydrolyzation of glycosidic bonds during the crushing and malaxation processes leads to the formation of aglycones, primarily in the non-aldehydic closed form [10]. The initial aglycone compounds (closed form I) are then transformed by hydrolysis and keto-enol tautomerism into the open forms I and II (dialdehyde) and by a 1,4-Michael addition into the closed forms II and III (mono-aldehydes) [10,11]. These transformations can take place because the pH of the olive paste is approximately five and the compounds undergo an isomerization reaction with the acid in the water phase. Additionally, oleacein (OLE) and oleocanthal (OLC), also with open and closed forms, are generated through a decarboxylation step. The proposed processes of biotransformation and isomerization are depicted in Figure 1.

Those phenolic compounds are the responsible of some EVOO health effects [12]. OLC is found to be a nonsteroidal anti-inflammatory compound, with effect similar to ibuprofen [13]. Furthermore, consumption of EVOO rich in OLC is associated with an anti-platelet effect, which may affect the prevention of cardiovascular diseases [14]. OLC also plays a remarkable role against neurogenerative diseases, primarily it reduces oxidative stress and protects against neuronal apoptosis [15]. It plays specific roles against Alzheimer’s Disease, it inhibits β-amyloid formation, reduces its toxicity and enhances the clearance from the brain [16,17,18]. On the other hand, OLE prevents cardiovascular diseases by inhibiting LDL oxidation and preventing neutrophil adhesion [19,20]. OLE also has anti-cancer effect promoting apoptosis in leukemia and skin cancer cells [21,22]. LIG also has shown anti-cancer effect, against breast cancer cells inducing apoptosis [23] and inhibiting c-MET signaling and then having antimigratory activity [24]. Finally, OLA has proven to reduce the levels of total cholesterol, LDL, and triglycerides and to increase HDL and liver antioxidant enzymes in vivo [25].

SEC concentration in EVOO depends on different agronomical and technical factors. For instance, the variety is the most affecting factor [26]. For example, varieties such as the Spanish “Picual” or the Italian “Coratina” contain high concentrations in SEC [26,27]. Additionally, there are some Ivory-wild varieties that produce an EVOO very rich in SEC [28]. Another factor that affects SEC concentration is the maturity stage of the olives. The different enzymatic activities of the olive varieties cause the phenolic profile to vary distinctly during the ripening [27]. Moreover, technical factors also play an important role during EVOO extraction, an increase in the malaxation temperature will lead to an increase in enzymatic activity during EVOO extraction [29]. In addition, an increase in malaxation time will increase the time those enzymes are working, having more reaction from LIG and OLA to OLC and OLE, respectively, then increasing OLC and OLE concentration and decreasing LIG and OLA [30]. Finally, the type and diameter of sieve also affect the oil composition [31]. Thus, an accurate analysis of those compounds could shed light on how agronomical and technical factors affect the bioactive components of EVOO.

Different methods are available for the analysis of EVOO phenolic compounds [32,33], but liquid chromatography coupled to mass spectrometry (MS) has proven to be the best in terms of selectivity and limits of detection (LOD) and quantification (LOQ) [34]. Colorimetric methods have been well correlated with individual phenols, although they only give an idea of the total amount and not of the phenolic profile of the oil [35]. However, during the chromatographic analysis, the open-structure forms of the four main SEC of EVOO can undergo keto-enol tautomerism, and monohydrates, methyl hemiacetals and dimethyl acetals can be generated when the sample is prepared with water and methanol (MeOH). Therefore, chromatographic analysis of those SEC has several impediments. In addition, the open-ring forms I and II (dialdehyde), closed form I (non-aldehyde) and closed forms II and III (mono-aldehyde) can be detected when OLA and LIG are in contact with a protic solvent such as water [10,36,37]. SEC can also form acetals and hemi-acetals with MeOH and water [38]. Curiously, the methyl hemi-acetal of OLC has been previously detected in EVOO but not identified, being reported as unknown [39].

The methods of Suarez et al. (2008) resulted in wide and jagged peaks for OLC and OLE [40]. To block the carbonylic species and avoid the tautomeric equilibrium and interaction with solvents, Di Donna et al. (2011) proposed carrying out in situ chemical derivatization of the aldehyde functions with methoxyamine before the analysis of OLC and OLE with high-performance liquid chromatography (HPLC) coupled with mass spectrometry in tandem (MS/MS), but this extra step resulted in a more complicated and expensive method [41]. Alternatively, Karkoula et al. (2012) developed a quantitative ^1^H-NMR method for the analysis of the dialdehyde forms of OLC and OLE [42], which was later extended to the analysis of the closed monoaldehyde forms of the aglycones [43]. However, a large amount of EVOO (5 g) and solvents (50 mL) are required to ensure a sufficient quantity of analytes in the sample, and the extract cannot be used for the analysis of the other phenolic compounds. Another disadvantage is that this method also needs an external calibration curve, as the extraction is not complete, requiring then large amounts of pure compounds, although this calibration curve is just needed once, as the NMR response is constant. Furthermore, the method cannot quantitate all isoforms of LIG and OLA, just the closed monoaldehyde forms. (closed forms II Figure 1). Diamantakos et al. (2015) determined that the ratio of the open dialdehyde forms and the open monoaldehyde form (the corresponding enolic form) was 1:1:2, respectively [37]. Later, the same authors used the 1:1:2 ratio to calculate also the concentration of these form integrating the enol peak of the open monoaldehyde form [44]. However, the reagent and time-consuming problem of the extraction required for the NMR analysis is not solved.

Sánchez de Medina et al. (2017) developed a method to determine OLC and OLE, but not the other EVOO SEC [38]. In addition, in later experiments, they also quantitate OLA and LIG, but without method validation [45]. Moreover, Celano et al. (2018) studied the SEC transformations during the extraction and chromatographic process and validated a method for the quantitation of SEC through ultra-high performance liquid chromatography (UHPLC) coupled to a UV detector. However, this approach is time-consuming (27 min) and the LOD and LOQ are high [46]. Finally, a direct method for the analysis of SEC by UHPLC coupled to MS/MS, minimizing the generation of acetals and hemiacetals, was recently validated. Nevertheless, a separate extraction process was required, as the extraction method proved unsuitable for the other phenolic compounds [47]. Furthermore, a ternary pump and a chromatographic instrument adequate for strong organic solvents, not always available, were also necessary.

Herein we report the development and validation of a simple analytical method based on UHPLC coupled with electrospray ionization and MS/MS (UHPLC-ESI-MS/MS) for the analysis of the four major SEC compounds in EVOO, able to coelute their isomers and achieve well-shaped peaks. Matrix-matched calibration was used, employing the same extraction process as for the other phenolic compounds in EVOO and a defatting step. The matrix effect of the oil and the analyte recoveries were also assessed.

## 2. Materials and Methods

### 2.1. Reagents

OLC (≥95% purity) was purchased from Merck (Darmstadt, Germany), and OLE (≥90% purity) and OLA (≥95% purity) from Toronto Research Chemicals (North York, ON, Canada). MeOH, acetonitrile (ACN) and formic acid were acquired from AppliChem, Panreac Quimica SLU (Barcelona, Spain). Hexane, 4-hydroxybenzaldehyde, chloroform-*d*, tetramethylsilane and gentisic acid were purchased from Sigma-Aldrich (St. Louis, MO, USA). 3-(4-hydroxy-3-methoxyphenyl)propionic acid was purchased from Fisher Scientific (Waltham, MA, USA) and cyclohexane from Carlo Erba (Madrid, Spain). Ultrapure water was obtained using a Milli-Q purification system (Millipore, Bedford, MA, USA).

### 2.2. SEC Extraction for UHPLC-ESI-MS/MS Analysis

SEC were extracted by a liquid-liquid extraction following the procedure proposed by Capriotti et al. (2014) [48]. Briefly, 0.5 g of EVOO was dissolved in 1 mL of hexane in a 10 mL centrifuge tube and shaken for 30 s. The phenolic compounds were extracted as follows: 2 mL of a solution of MeOH:water (4:1 *v*/*v*) was added to the tube and stirred for 30 s. The resulting emulsion was centrifuged at 3000 rpm and 4 °C for 3 min. After separation of the hexane phase, the methanolic-aqueous extract was washed with hexane. To ensure the complete removal of SEC from the hydrophobic phase, a second extraction of phenolic compounds was carried out from the hexane phase with MeOH:water (4:1 *v*/*v*), using the aforementioned conditions. The combined methanolic-aqueous extracts were evaporated under a nitrogen curtain and reconstituted with 800 μL of MeOH:water (80:20 *v*/*v*), filtered with polytetrafluoroethylene syringe filters (0.2 µm), transferred to an amber glass vial, and stored at −80 °C until analysis. All procedure was done under UV-filtered light to avoid SEC photooxidation.

The calibration curve was prepared in refined olive oil, which was spiked with the standards before extraction at the following concentrations: 1, 2, 5, 8, 10, and 20 mg·kg^−1^.

### 2.3. UHPLC-ESI-MS/MS Analysis

The analysis was carried out using a UHPLC Acquity system equipped with a binary pump and autosampler (Waters, Milford, MA, USA), coupled to an API3000 triple quadrupole mass spectrometer (ABSciex, Framingham, MA, USA) equipped with a TurboIonspray source operating in negative mode. An Acquity UPLC^®^ BEH C18 column (2.1 × 50 mm, i.d., 1.7 µm particle size) with an Acquity UPLC^®^ BEH C18 pre-column (2.1 × 5 mm, i.d., 1.7 µm particle size) (Waters Corporation^®^, Dublin, Ireland) were used for the main SEC separation. The mobile phases consisted of MeOH with 0.1% of formic acid (A) and water with 0.1% of formic acid (B) and the flow was constant at a rate 0.6 mL·min. The gradient was as follows: t = 0 min, 100% (A); t = 2 min, 100% (A); t = 4.75 min, 46.4% (A); t = 4.9 min, 0% (A); t = 5.9 min, 0% (A); t = 6 min, 100% (A); t = 6.5 min, 100% (A). The injection volume and column temperature were 5 µL and 50 °C, respectively.

Ionization, in negative mode, was achieved using electrospray ionization (ESI) and all the compounds were monitored in the multiple monitoring mode (MRM) with the following settings: capillary voltage, −4000 V; nebulizer gas (N_2_), 10 (arbitrary units); curtain gas (N_2_), 12 (arbitrary units); and drying gas (N_2_) heated to 450 °C at flow rate 8000 c/min. The declustering potential, focusing potential, collision energy and entrance potential are shown in Table 1. The system was controlled by Analyst version 1.4.2 software supplied by ABSciex, the chromatograms were integrated with this same software.

### 2.4. Validation of the UHPLC-ESI-MS/MS Method

The method was validated following the AOAC guidelines for dietary supplements and botanicals [49]. A matrix-matched calibration was selected, as previous experiments show low analyte recoveries [40]. The standards used were OLC, OLE, and OLA, but not LIG, as it is not commercially available. Selectivity, linearity, accuracy, repeatability, the carry-over between samples and the LOD and LOQ were tested. Regarding the selectivity, the ability to distinguish the analyte from other substances was indicated by an absence of the respective peaks at the same retention time as the corresponding standards in trace chromatograms obtained in multiple monitoring mode.

For assessing the cleaning step of the chromatographic method, the carry-over was evaluated. The carry-over is the increment of the signal of a peak due to residual analyte from preceding sample that remains in the instrument [50]. As AOAC guidelines do not have a protocol to evaluate the carry-over, the guideline on bioanalytical method validation from the European medicines agency was followed [50]. Hence, a blank sample was injected after the highest peak of the calibration curve (20 mg·kg^−1^) and the carry-over should not be greater than 20% of the LOQ.

The linearity was evaluated by developing calibration curves using refined olive oil spiked with the analytes before the extraction, with 6 concentration points ranging from 1 to 20 mg·kg^−1^ and five replicates for each point. The residuals were then tested through a residual plot. The R^2^ was also calculated, as were the accuracies of each point of the curve, based on the ratio between the calculated concentration and the spiked concentration; following AOAC guidelines, accuracies between 80 and 120% were accepted as valid. When necessary, weighted regression was applied to improve the accuracy [51]. For calculating the curves with weighting, and without it, and for the residual plot test R version 4.0.3 (The R foundation) with RStudio version 1.3.1 (Rstudio, PBC) software was used. For the rest of the calculus, Excel 2017 (Microsoft SA) software was used.

To determine the repeatability of the method, both intra- and inter-day repeatability were tested. Five replicates of a low-, intermediate- and upper-point of the curve (1, 5, and 10 mg·kg^−1^) were injected three times on the same day for the intra-day and three times on three different days for the inter-day repeatability. The relative standard deviations (RSD) for the inter- and intra-day experiments were calculated. All the values lower than RSD = Concentration^−(0.15)^ were accepted. Those values are lower than 8% for 1 mg·kg^−1^, 7% for 5 mg·kg^−1^ and 6% for 10 mg·kg^−1^.

The LOD is the smallest concentration of the analyte that can be distinguished from the blank and by definition is 3 times the standard deviation of the blank. The LOQ is the smallest amount or concentration of an analyte that can be determined with acceptable reliability and corresponds to 10 times the standard deviation of the blank. The LOD and LOQ were determined using the lower point of the curve, calculating the signal-to-noise ratio of that point, and extrapolating the concentration with a signal-to-noise ratio of 3 and 10, respectively. Then each analyte was prepared at the LOD and LOQ concentration to verify the obtained value.

### 2.5. Matrix Effect and Recovery

The matrix effect and the recovery of the developed method were assessed following the procedure of Matuszewski et al. (2003) [52]. The matrix effect is the change of the analyte signal due to coeluted substances, whilst the recovery is the percentage of the analyte that remains after the extraction. Three different calibration curves were prepared, two of them using refined olive oil spiked with the standard SEC compounds before and after the extraction process, named “spiked-before” and “spiked-after” curves (SBC and SAC, respectively). The third curve was generated by directly diluting the standards in the reconstitution phase, namely, the reconstitution phase curve (RPC). By comparing the SBC and SAC, it is possible to evaluate the recovery of the extraction. Whereas the matrix effect can be assessed by comparing the SAC and RPC, as the interferences form the matrix are not present in the RPC.

## 3. Results and Discussion

### 3.1. Analytical Method Development

The chromatographic conditions were established as in a previous study [38]. Different percentages of formic acid (from 0.02% to 1%) in both mobile phases were tested to improve the peak shape and ionization of the SEC. The best ionizations were achieved with 0.1% of formic acid in both water and MeOH. The ionization parameters were then optimized for each compound to obtain the highest signal through direct infusion of the analytes at 1 mg·L^−1^. The parameters optimized were the declustering potential, focusing potential, collision energy, and entrance potential. Finally, the dwell time was optimized to obtain a smooth peak. The results are shown in Table 1.

**Figure 2:**
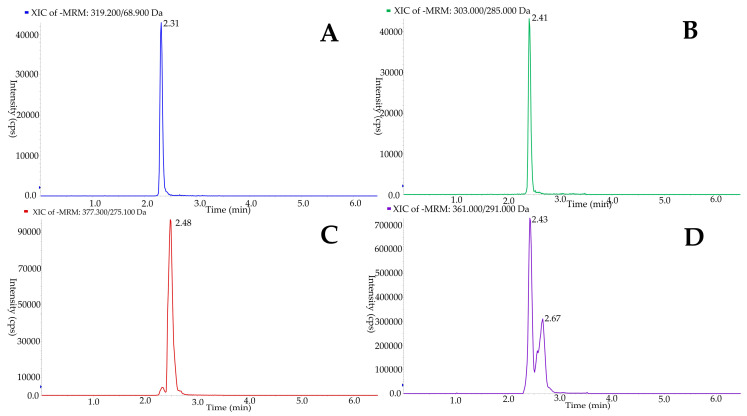
Example chromatogram of oleacein (**A**), oleocanthal (**B**), oleuropein aglycone (**C**), and ligstroside aglycone (**D**), the latter from a commercial “Picual” EVOO.

As already mentioned, the coexistence of open- and closed-structure forms of SEC compounds hinders their chromatographic analysis. However, we improved the gradient to minimize the number of peaks for each compound and obtained thin and symmetric peaks. These new chromatographic conditions allowed us to coelute the different isomers together, all of which could thus be quantified at the same time. An example of a chromatogram for each analyte is shown in Figure 2. OLE and OLC isoforms were eluted in one peak, whereas OLA and LIG still produced more than one, although with acceptable accuracy.

Finally, when analyzing oil samples with a reverse phase column, sometimes the chromatographic conditions cannot properly clean the column and cross-contamination of samples can occur. To prevent this, Luque et al. (2019) implemented a cleaning step based on ACN and tetrahydrofuran, requiring a ternary pump system and an HPLC instrument prepared for strong solvents, which are not always available [47]. In our study, the chromatographic cleaning step was confirmed by testing the carry-over in a blank sample injected right after the most concentrated point of the calibration curve (20 mg·kg^−1^). The carry-over for OLE was null, in the case of OLC and OLA, despite being a small peak, it was less than 20% of the LOQ. The chromatograms are showed in Figure S1. As the extraction method includes a defatting step with hexane, the use of strong organic solvents was not needed to avoid carry-over.

Additionally, the use of an internal standard was tested to minimize errors. The best internal standards for a MS quantitation method are compound analogues isotopically labeled with deuterium (D) or ^13^C, but OLA, LIG, OLE, and OLC derivatives are not commercially available. Therefore, compounds structurally similar to our target analytes were tested as internal standards, such as gentisic acid and 3-(4-hydroxy-3-methoxyphenyl)propionic acid. Those were tested at 5 mg·kg^−1^, corresponding to an intermediate concentration of the calibration curve. However, the resulting accuracies were lower than without the use of internal standards.

### 3.2. Analytical Method Validation

The method was assessed in terms of linearity, selectivity, accuracy, repeatability, and LOD and LOQ (results shown in Table 2). The selectivity was visually checked in the chromatograms, which showed no other analytes except those of interest.

The linearity range was examined visually in the residual plot. In the case of OLC and OLA, a 1/x^2^ and 1/x weighting was used, respectively, to improve the accuracy for the low concentration points [51]. Then, as the curve was built with a weighted least squares method, the linearity was checked through the standardized residual plots (depicted in Figure S2). All the three analytes (OLC, OLE, and OLA) showed linearity in the tested range between 1 and 20 mg·kg^−1^. Other studies report linearity from 3 to 200 µg·kg^−1^ for OLE and LIG, from 1 to 100 µg·kg^−1^ for OLA, and from 12 to 800 µg·kg^−1^ for OLC [47]. However, those margins are far below the usual concentration of these compounds in EVOO [45].

The LODs were in the order of 20 µg·kg^−1^ and LOQs approximately 100 µg·kg^−1^, which are sufficiently low to quantitate the SEC in EVOO [45]. The limits are higher than those reported by Luque et al. who reported a LOD of about 1 µg·kg^−1^ and LOQ of about 3 µg·kg^−1^, with even lower values for OLA (0.3 µg·kg^−1^ and 0.9 µg·kg^−1^, respectively) [47]. An explanation for these different results is that Luque-Muñoz et al. were using a more sensitive mass spectrometer (a Xevo TQS tandem quadrupole mass spectrometer from Waters) than the one used in this work. However, the LOD and LOQ of our method were far lower than those achieved with the ^1^H-NMR procedure published by Karkoula et al. (2014), in which the LOD was 1 mg·kg^−1^ and the LOQ was 10 mg·kg^−1^ for all compounds [43]. These high values are due to the analytical technique, as NMR is less sensitive than MS [53]. The LOD and LOQ obtained here are also lower than those reported by Suarez et al. [40]. Further comparison between methods is summarized in Table 3.

The accuracy of the method was within the accepted limits of the AOAC guidelines (80–120%) for all the concentrations and analytes. Other studies report similar results: the accuracies found by Luque et al. are all less than 15% and those of Karkoula et al. (2012) are less than 16% [42,47].

Lastly, both the intra and inter-day repeatability of the method were within the accepted margins established by AOAC guidelines, which stipulate that the maximum RSD should be 6% for 10 mg·kg^−1^, 7% for 5 mg·kg^−1^ and 8% for 1 mg·kg^−1^. Similarly, Luque et al. reported an RSD between 2.5 and 8.5% for OLC at 594 ng·mL^−1^ and 24.75 ng·mL^−1^, respectively [47]. In contrast, Sanchez de Medina et al. showed higher deviations, being 10% for OLC and 11% for OLE, although in this case the concentrations were not specified [38].

### 3.3. Matrix Effect and Recovery

Table 4 shows the results of the matrix effect and the recoveries for each concentration of the curves and compound, calculated as described above. The recoveries for the phenolic extractions were partial and varied according to the compound and concentration, ranging between 50 and 100%. Suarez et al. also found quite low recoveries for OLC (67%) and OLE (71%) [40].

Matrix effect was observed due to the substances coextracted and coeluted in the chromatography with the phenols. In the case of OLE and OLA, the matrix effect was less than 100%, indicating matrix suppression; the values ranged from 50 to 90% for OLE and 20 to 69% for OLA. On the contrary, matrix enhancing was observed for OLC, with values ranging from 75 to 150%.

To minimize the error due to the matrix effect and recoveries, internal standard method, or matrix-matched method are suitable, both make the matrix effect and the recoveries the same for the curve and the samples, although the internal standard method does not need a white matrix. In this study a matrix-matched external calibration was carried out to validate the method, it was possible to use this calibration method as the refined olive oil served as a blank matrix.

## 4. Conclusions

Simple, quick and with reasonable cost analytical methods are needed for the better understanding of our world. In this context, this publication presents a suitable method for the analysis of EVOO SEC, whose analysis has been a challenge since their characterization [54]. The best methods up to now were NMR methods, which despite presenting a lot of advantages, require a high time- and reagent-consuming extraction of the samples. Furthermore, there is an UHPLC validated method for EVOO SEC analysis, which uses a non-generic UHPLC and requires a different sample extraction than the one used for other EVOO phenolic compounds. Therefore, in this work, a quick (6.5 min) chromatographic method was developed and validated for the analysis of the main SEC compounds in EVOO, using the same extraction procedure employed for the other EVOO phenolic compounds, rendering it low time- and reagent-consuming. The linearity of the method was between 1 and 20 mg·kg^−1^, the accuracies were between AOAC accepted margins, and the LOD and LOQ relatively low (in the order of 20 and 100 µg·kg^−1^, respectively). The cleaning step of the chromatographic method was good as the carry-over was null. This method would provide new tools for a better understanding on how EVOO phenolic profile changes during extraction and due to agronomical conditions, such as olive variety or olive maturity stage. Finally, it would help future research to determine better the effect of EVOO phenolic profile on animal and human health.

## Figures and Tables

**Table 1:** Electrospray ionization parameters for the secoiridoids (SEC).

	OLE	OLC	OLA	LIG
MRM Transition	361/291	303/285	377/275	319/69
Declustering Potential	−40	−40	−45	−30
Focusing Potential	−170	−170	−140	−170
Entrance Potential	−5	−5	−5	−5
Collision Energy	−10	−10	−15	−30
Dwell Time (ms)	150	50	150	50
Retention time (min)	2.7	2.4	2.5	2.3

**Table 2:** Method validation parameters for OLE, OLC, and OLA.

		OLE	OLC	OLA
Calibration curve	Slope	27,987	33,800	82,737
	Interception	−19,765	−7875	−74,897
	R^2^	0.9991	0.9873	0.9907
	Weighting	none	1/x^2^	1/x
Limits	LOD (mg·kg^−1^)	0.0343	0.0186	0.0334
	LOQ (mg·kg^−1^)	0.114	0.0621	0.111
Accuracies	1 mg·kg^−1^	93%	107%	87%
	2 mg·kg^−1^	100%	89%	101%
	5 mg·kg^−1^	103%	97%	113%
	8 mg·kg^−1^	96%	95%	98%
	10 mg·kg^−1^	102%	100%	110%
	20 mg·kg^−1^	99%	114%	94%
Repeatability Relative Standard Deviation	Intraday 1 mg·kg^−1^	2.3%	2.5%	5.8%
	Intraday 5 mg·kg^−1^	1.9%	2.7%	1.8%
	Intraday10 mg·kg^−1^	1.1%	5.7%	3.7%
	Interday 1 mg·kg^−1^	2.4%	3.3%	7.5%
	Interday 5 mg·kg^−1^	5.7%	4.1%	7.0%
	Interday 10 mg·kg^−1^	1.2%	6.0%	3.1%

**Table 3:** Parameter comparison between methods for oleacein (OLE), oleocanthal (OLC) and oleuropein aglycone (OLA).

	LOD (mg·kg^−1^)	LOQ (mg·kg^−1^)	R^2^	RSD (%) ^a^	EVOO (g)	Instrument	Time of Analysis (min)	Study
OLE	0.0343	0.114	0.999	1.8	0.5	UHPLC-ESI-MS/MS	6.5	Current study
	0.128	0.428	-	3.1	45	UPLC-ESI-MS/MS	40	[40]
	0.002	0.005	0.999	10	1	HPLC-ESI-MS/MS	10	[38]
	0.001	0.0033	0.995	1.9	1	UHPLC-ESI-MS/MS ^b^	7.5	[47]
	1	10	0.994	4.3	5	NMR	-	[42]
OLC	0.0186	0.0621	0.987	3.6	0.5	UHPLC-ESI-MS/MS	6.5	Current study
	0.072	0.244	-	3	45	UPLC-ESI-MS/MS	40	[40]
	0.004	0.01	0.992	11	1	HPLC-ESI-MS/MS	10	[38]
	0.004	0.012	0.994	4.4	1	UHPLC-ESI-MS/MS ^b^	7.5	[47]
	1	10	0.999	4.3	5	NMR	-	[42]
OLA	0.0334	0.111	0.991	3.8	0.5	UPLC-ESI-MS/MS	6.5	Current study
	0.0003	0.0009	0.996	2.5	1	UHPLC-ESI-MS/MS ^b^	7.5	[47]
	1	10	0.999	4.1	5	NMR	-	[43]

The parameters were chosen according to the criteria of the authors to help choose the most suitable analytical method. ^a^ Relative standard deviation within the same day of analysis, when it was calculated for more than one concentration; the mean of the different RSD is showed in the table. ^b^ The chromatographic instrument requires a ternary pump and to be prepared for strong organic solvents.

**Table 4:** Matrix effect and recoveries for OLE, OLC, and OLA.

		OLE	OLC	OLA
Matrix effect (%)	1 mg·kg^−1^	56	93	20
	2 mg·kg^−1^	50	76	27
	5 mg·kg^−1^	86	129	57
	8 mg·kg^−^^1^	79	123	51
	10 mg·kg^−1^	82	134	56
	20 mg·kg^−^^1^	93	152	69
Recoveries (%)	1 mg·kg^−^^1^	55	59	66
	2 mg·kg^−1^	98	96	88
	5 mg·kg^−1^	80	61	74
	8 mg·kg^−^^1^	96	62	106
	10 mg·kg^−1^	89	61	88
	20 mg·kg^−^^1^	94	60	93

## Data Availability

Data is contained within the article and Supplementary Material.

## Supplementary Materials

The following are available online at https://www.mdpi.com/article/10.3390/antiox10040540/s1, Figure S1: Chromatograms of a water injection after a sample. Figure S2: Residual plot of the different calibration curves.

## Abbreviations

ACN: acetonitrile; ESI: Electrospray ionization; EVOO: Extra-virgin olive oil, HPLC: high-performance liquid chromatography; LIG: ligstroside aglycone; LOD: limit of detection; LOQ: limit of quantification; MeOH: methanol; MRM: multiple monitoring mode; MS: mass spectrometry; MS/MS: mass spectrometry in tandem; OLA: oleuropein aglycone; OLC: oleocanthal; OLE: oleacein; RPC: reconstitution phase curve; RSD: relative standard deviation; SAC: spiked-after curve; SBC: spiked-before curve; SEC: secoiridoids; UHPLC: ultra-high performance liquid chromatography; UV: ultraviolet.
