# Supplementary material for: Total Analysis of the Major Secoiridoids in Extra Virgin Olive Oil: Validation of an UHPLC-ESI-MS/MS Method
Source: Antioxidants (Basel). 2021 Mar 30;10(4):540. doi: 10.3390/antiox10040540 (PMC8066082; doi:10.3390/antiox10040540)

**Suplemmentary material**


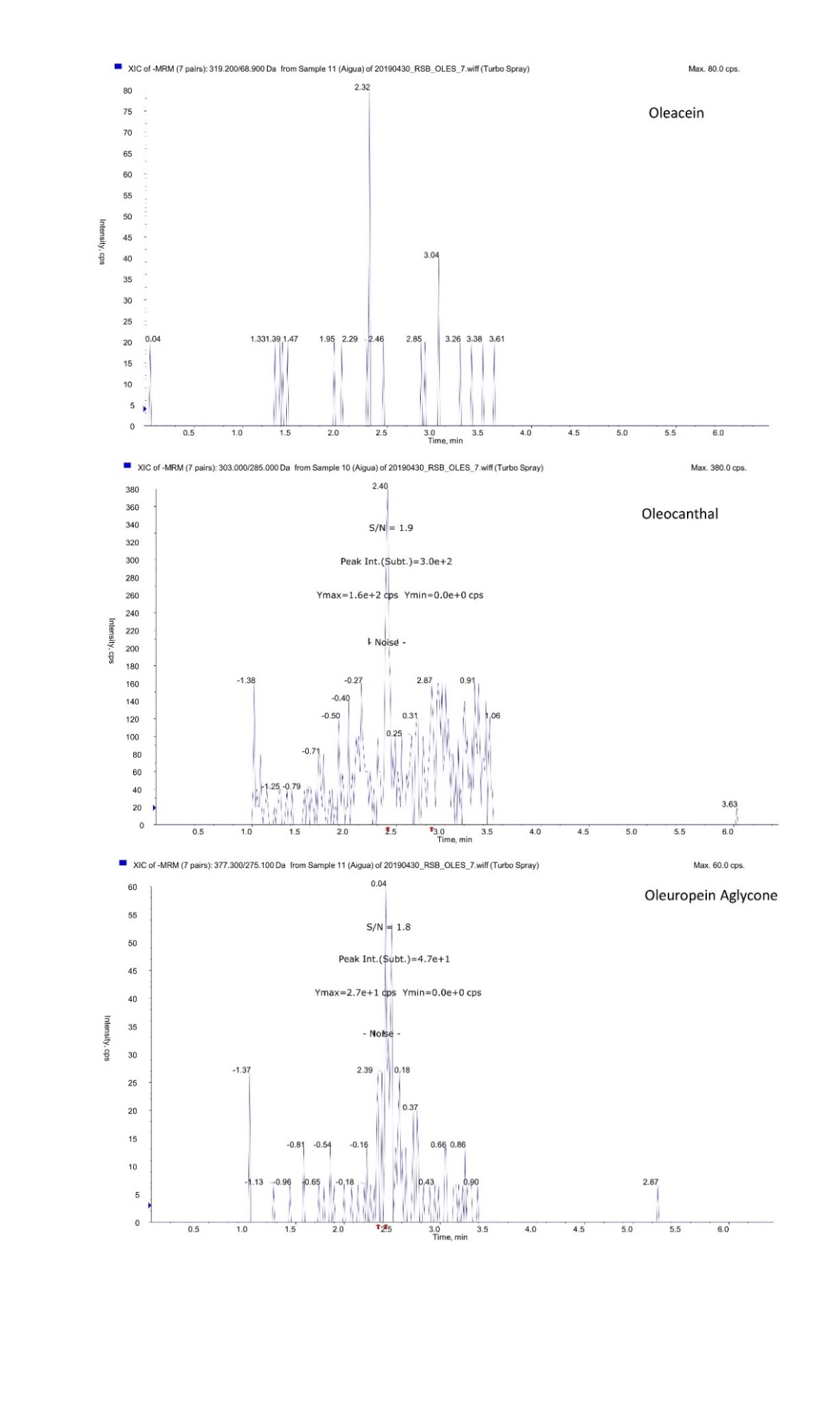
**Figure S1**: Example chromatograms of a water injection after three samples. Oleacein, Oleocanthal, Oleuropein aglycone and Ligstrosid Aglycone

**Figure S2**: Residual plot of the different calibration curves.


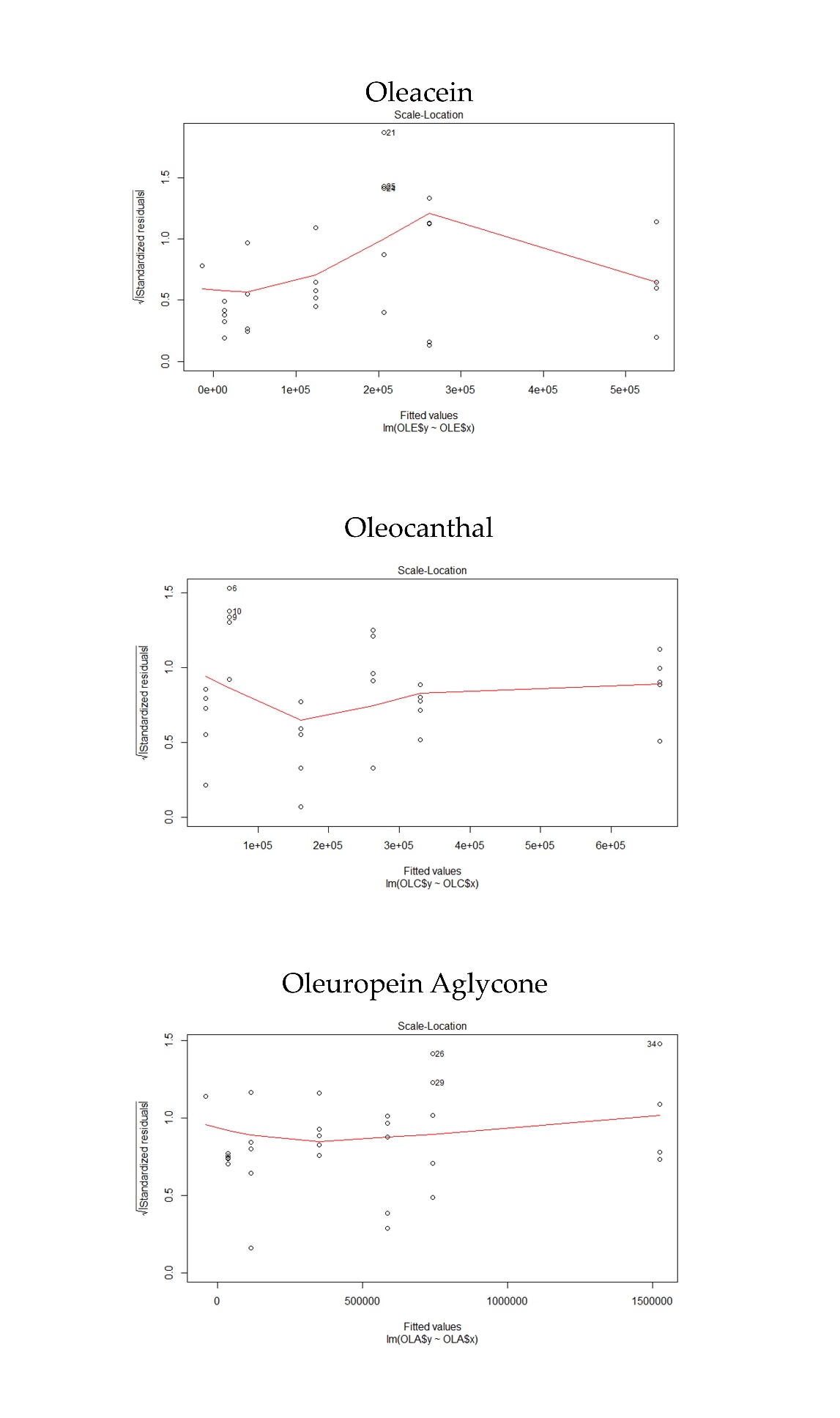

Supplement: Supplementary file 1 [file antioxidants-10-00540-s001.zip › supplementary_material.docx]
