# Supplementary figures and images for: Total Analysis of the Major Secoiridoids in Extra Virgin Olive Oil: Validation of an UHPLC-ESI-MS/MS Method
Source: Antioxidants (Basel). 2021 Mar 30;10(4):540. doi: 10.3390/antiox10040540 (PMC8066082; doi:10.3390/antiox10040540)

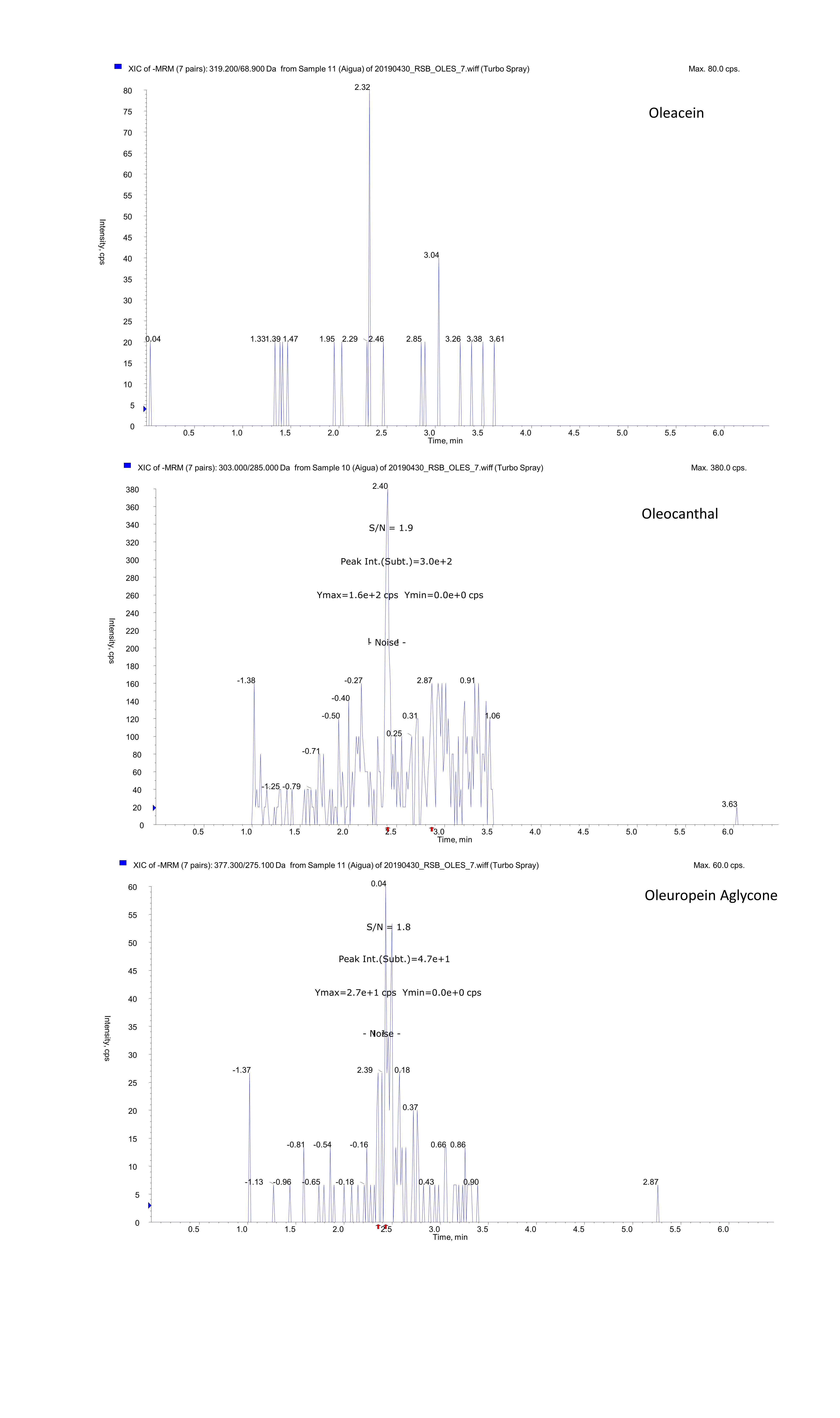

Supplement: Supplementary file 1 [file antioxidants-10-00540-s001.zip › figureS1.jpg]

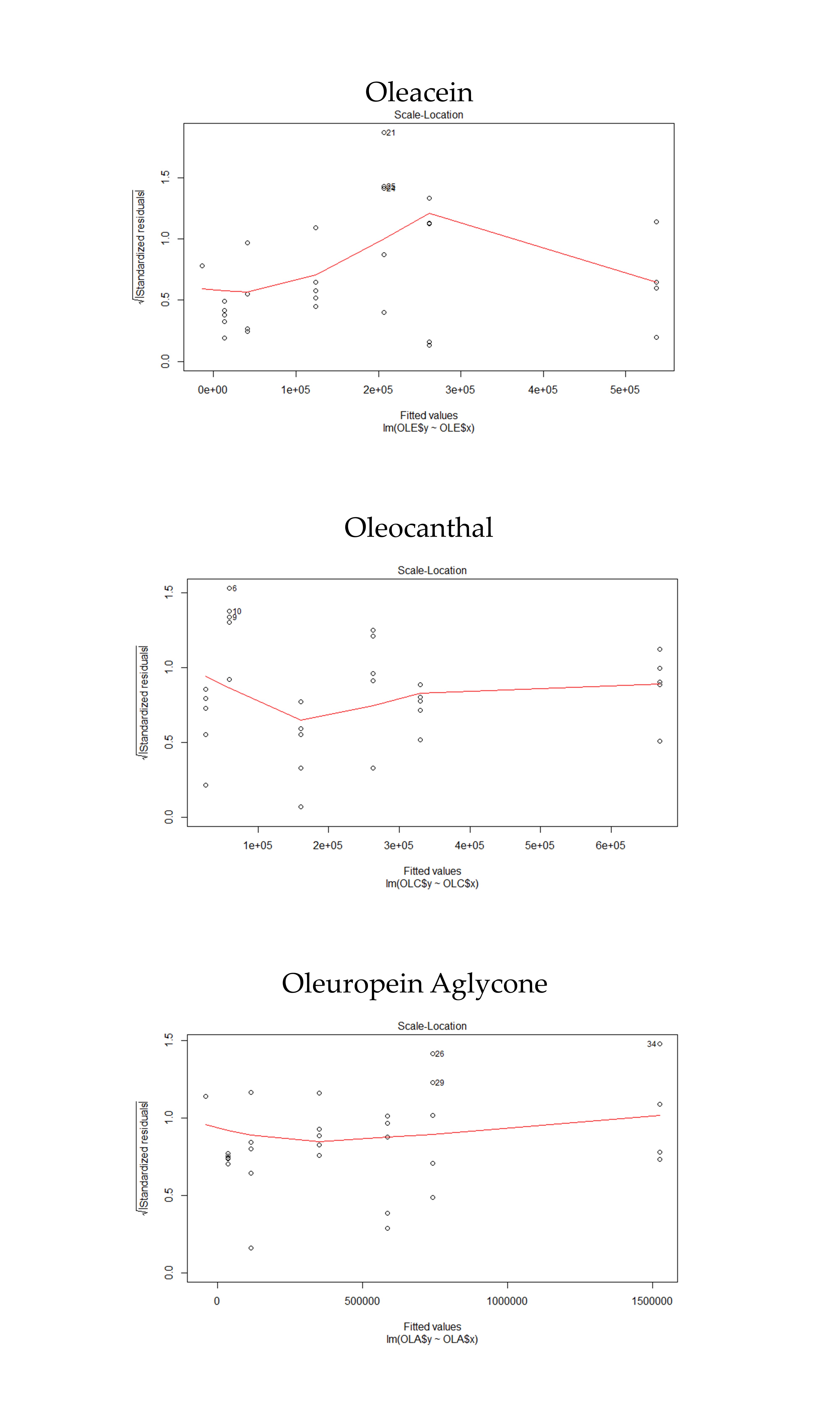

Supplement: Supplementary file 1 [file antioxidants-10-00540-s001.zip › figureS2.jpg]
